# Optimizing illumination in the greenhouse using a 3D model of tomato and a ray tracer

**DOI:** 10.3389/fpls.2014.00048

**Published:** 2014-02-18

**Authors:** Pieter H. B. de Visser, Gerhard H. Buck-Sorlin, Gerie W. A. M. van der Heijden

**Affiliations:** ^1^Department of Greenhouse Horticulture, Wageningen University and Research CentreWageningen, Netherlands; ^2^Institut de Recherche en Horticulture et SemencesAGROCAMPUS OUEST, Angers, France; ^3^Biometris, Wageningen University and Research CentreWageningen, Netherlands

**Keywords:** photosynthesis, greenhouse crop, HPS, LED, light distribution, LUE

## Abstract

Reduction of energy use for assimilation lighting is one of the most urgent goals of current greenhouse horticulture in the Netherlands. In recent years numerous lighting systems have been tested in greenhouses, yet their efficiency has been very difficult to measure in practice. This simulation study evaluated a number of lighting strategies using a 3D light model for natural and artificial light in combination with a 3D model of tomato. The modeling platform GroIMP was used for the simulation study. The crop was represented by 3D virtual plants of tomato with fixed architecture. Detailed data on greenhouse architecture and lamp emission patterns of different light sources were incorporated in the model. A number of illumination strategies were modeled with the calibrated model. Results were compared to the standard configuration. Moreover, adaptation of leaf angles was incorporated for testing their effect on light use efficiency (LUE). A Farquhar photosynthesis model was used to translate the absorbed light for each leaf into a produced amount of carbohydrates. The carbohydrates produced by the crop per unit emitted light from sun or high pressure sodium lamps was the highest for horizontal leaf angles or slightly downward pointing leaves, and was less for more upward leaf orientations. The simulated leaf angles did not affect light absorption from inter-lighting LED modules, but the scenario with LEDs shining slightly upward (20^°^) increased light absorption and LUE relative to default horizontal beaming LEDs. Furthermore, the model showed that leaf orientation more perpendicular to the string of LEDs increased LED light interception. The combination of a ray tracer and a 3D crop model could compute optimal lighting of leaves by quantification of light fluxes and illustration by rendered lighting patterns. Results indicate that illumination efficiency increases when the lamp light is directed at most to leaves that have a high photosynthetic potential.

## INTRODUCTION

The spatial distribution of assimilation lights in greenhouse horticulture, especially in countries with a deficiency in natural sunlight, is a controversially debated topic (Hovi-Pekkanen and Tahvonen, 2008; Trouwborst et al., 2010; de Visser and Buck-Sorlin, 2011). However, there is general agreement that lamp type, density, positioning and orientation with respect to plant orientation are important and that an optimization of these factors can help improve crop light interception and thus reduce energy costs. Finding the optimal solution experimentally is nearly impossible due to the large number of possible combinations and the high financial, and time investment per experiment. Computer-aided design coupled with 3D modeling of plants and light distribution could be a more rapid and cost-effective solution in order to test the effect of possible lamp configurations in the greenhouse on crop light interception. Software tools (e.g., computer aided design – CAD) that enable 3D visualization of objects in a 3D scene, including the simulation of the trajectories of light rays using ray tracing methods, are well established and tested. Commonly used methods to simulate light distribution in plant canopies are Monte Carlo ray tracing (Veach, 1997) and nested radiosity (Chelle and Andrieu, 1998). The exponential increase in computing power also allowed the further development of simple crop models into more elaborate versions, so-called functional-structural plant models (FSPM; Vos et al., 2010). FSPM refers to a paradigm for the description of a plant by creating a (usually object-oriented) computer model of its structure and selected physiological and physical processes, at different hierarchical levels: organ, plant individual, canopy (a stand of plants), and in which the processes are modulated by the local environment. With respect to greenhouse crops FSPMs for tomato (Sarlikioti et al., 2011a,b), cut rose (Buck-Sorlin et al., 2011) cucumber (Kahlen et al., 2008; Kahlen and Stützel, 2011; Wiechers et al., 2011) and *chrysanthemum* (de Visser et al., 2007) have been devised. Although in greenhouse practice a small number of 3D models of greenhouse structure including lamps are available, the model by de Visser et al. (2012) is to our knowledge the only one that considers the greenhouse interior (lamps, slabs) as well as the 3D structure and physiology of the plants. The reported model simulates the light distribution of natural (diffuse and direct) daylight and artificial light within a realistic 3D representation of a crop.

de Visser et al. (2012) simulated some energy saving light strategies by optimization of position of local LED lights in the crop, but the options of lamps with specific angles to better illuminate the leaves at angles changing with age were not addressed. Moreover, answers are needed on effects of leaves oriented toward the path, as raised by Sarlikioti et al. (2011b) and whether leaf positions are equally important for High-pressure sodium (HPS) and LED lamps. With the upcoming technology of LEDs as a (partial) alternative for HPS lamps, growers have an instrument to adapt the angles of the LED modules to account for local leaf angles. The 3D modeling can be used to find promising set-ups of LED lighting in a tomato crop that may result in increased light absorption and crop growth, concomitantly supporting a more energy saving lighting strategy.

## MATERIALS AND METHODS

The GroIMP interactive modeling platform, initially developed and described by Kniemeyer (2008) and maintained by the University of Göttingen (Germany), was used for the simulations. A virtual greenhouse was constructed within GroIMP on basis of an existing greenhouse compartment at the Improvement Center, Bleiswijk, The Netherlands, by explicitly considering the positions, shapes and optical properties of all its constituting objects (see below) in a 3D scene. The light distribution at a given time step was then computed by the GroIMP radiation model, which is based on an inversed Monte Carlo path tracer, similar to the one used by Cieslak et al. (2008). Sunlight was modeled as a direct and a diffuse component, depending on the 10-year average recorded outside light level. Diffuse light came from a sky object consisting of 72 directional lights arranged in a hemisphere around the greenhouse, whilst direct sun light was provided by a single directional light. The power of both light sources, as well as the position of the sun was a function of latitude, day of year, and time of day (Goudriaan and van Laar, 1994).

Optical properties of all greenhouse objects as well as leaves entailed reflection, transmission and absorption of the fraction of photosynthetic radiation (PAR) generated by a light source, and were measured on subsamples with a Lambda 1050 spectrophotometer (Perkin-Elmer Inc) coupled to a snap-in light integrating sphere.

Net photosynthesis was simulated for each leaflet on the basis of absorbed light, air temperature and CO_2_ according to Kim and Lieth (2003), with a leaf-age-depended value for Jmax, the potential rate of electron transport (in μmol electrons m^-^^2^ s^-^^1^). Measured light-response curves (3 heights in the crop, *n* = 3, 10 PAR levels from 0 to 2000 μmol m^-^^2^ s^-^^1^, measured with a Licor 6400 by gas exchange) at 700 ppm CO_2_ (annual average in greenhouse air) for winter and summer were used to calibrate parameters *f* (spectral correction factor, dimensionless) and Jmax in the photosynthesis model by reducing least square differences between modeled and observed photosynthesis.

**FIGURE 1:**
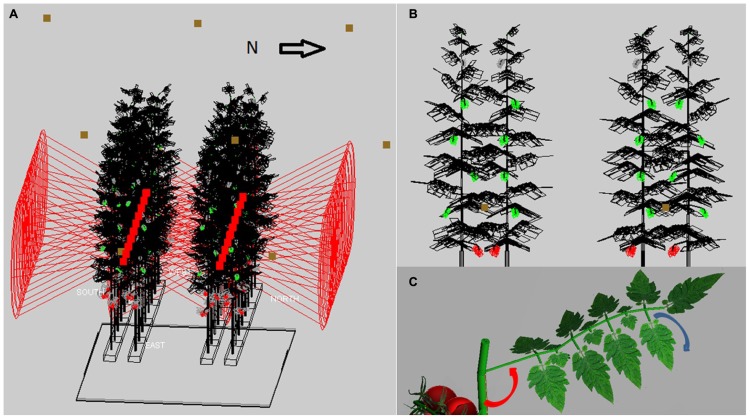
**The modeled 3D scene of the tomato crop: (A)** A crop of 32 plants (wireframe with black leaves, green unripe trusses and red ripe trusses) on two double rows of slabs at 0.8 m above the floor, with LED (red squares) inside the double row at 2.4 m height and HPS lamps (brown squares) at 1.5 m above the crop, **(B)** cross-section of four plants with leaf angles increasing with height (LED position at brown square), **(C)** rendered image of a single leaf with its rachis at a specific angle (red arrow) with the main stem, and consisting of leaflets that hang down by 30-50° (see blue arrow) relative to the horizontal.

The virtual greenhouse consisted of a glass roof, side walls, floor, energy-saving screen, gutters, assimilation lamps, and a crop consisting of static virtual plants (**Figure 1A**). The light pattern emitted by the HPS lamps of 1000 W concisely matched (per 10° interval) that of a SON-T in a wide angle reflector (data from Hortilux ©), see Buck-Sorlin et al. (2009) for details. The HPS lamps were placed in a grid of 6 m (in row direction) × 2.5 m (across rows) at 1.5 m above top of the crop. The emission pattern of LED light was simulated using simple, horizontally shining spotlights which had an opening angle similar to what has been measured on the 150 cm, commercially available, production module RB (data from Philips ©), and emitted PAR was calibrated to 60 μmol m^-^^2^ s^-^^1^ greenhouse. The strings with LEDs were at a height of 2.1 m above the ground, LEDs were 40 cm horizontally apart within each double plant row and placed in row direction. Ca. 3 leaves were situated below the LEDs which is regularly occurring in practice. The crop was represented as a static structure, corresponding to measurements on a tomato crop cv. Kommeet in our research facilities in Bleiswijk, The Netherlands: for the HPS and LED scenarios in winter, as measured on six plants on January 11th in 2011, for the sunlight scenarios in summer in another crop as measured on August 9th in 2011. The average value and its variation of leaf angles, length, width, internode length, and phyllotaxis were measured with ruler and protractor. The data were incorporated in GroIMP as average values per phytomer and the associated random variation, using a set of growth rules that created 32 (summer) or 36 (winter) phytomers per plant, of which eight were trusses and the rest consisted of leaves (**Figure 1B**). Each leaf was composed of 15 leaflets of a fixed geometry, yet their size increased in proportion to the length of the terminal leaflet of the composite leaf (**Figure 1C**). The modeled scene of 4 by 3.2 m ground area consisted of 32 plants with their lowest leaf oriented at a random azimuthal direction, an observed phyllotaxis of 130° between leaves, leaf angle of 90° (i.e., horizontal position) for lowest leaves and becoming slightly higher, maximizing at 130° for upper leaves ( **Figures 1B,C**). Plants were placed on slabs at 0.8 m above the floor, and pairs of slabs, with internal distance 0.4 m, were divided by a path, giving 1.6 m distance from center to center between slab pairs (**Figure 1A**). On a slab, plants were 0.4 m apart, and had only one stem without a split. Crop density was 2.5 stems per m^2^ ground floor; the top of the canopy was situated at maximally 4.5 m above the floor. Plant rows were oriented east-west as observed. An infinite canopy was simulated by placing perfect mirrors around the scene.

### SCENARIOS

Based on the above described default setup, different scenarios were examined to gain insight in the influence of plant architecture and light source specifics on crop light absorption and photosynthesis:

(1) Leaves were tilted by angles of +30% of default (i.e., from default 90 to 117° (lowest leaves) and from 130 to 169° (upper leaves), 0 and -30% (runs a, b, and c) relative to default, combined with different light sources: (1.1) daily course on June 21st of 10-year averaged sun light (70% direct, 30% diffuse), (1.2) HPS top light (130 μmol m^-^^2^ s^-^^1 ^PAR), (1.3) LED inter-lighting (60 μmol m^-^^2^ s^-^^1^ PAR)
(2) Leaves were forced to orientate toward the path (2a) or parallel to the slab (2b), combined with LED inter-lighting. Each of the leaf directions is imposed with a uniform continuous random variation between -10° and +10° in the horizontal plane, i.e., left and right of the main leaf direction.
(3) Inter-lighting with LED modules illuminating the plants at different angles [LEDs heading from upward by 30° (scenario 3a) to downward -30° (3f) in steps of 10°]

Each scenario was replicated five times, and at each run leaf angles were randomly varied in vertical direction between -10° and +10° of the measured angle, and each plant was turned around its axis randomly between -180 and +180°. Each run took ca. 10 s on a standard PC. Apart from percentage of emitted light per light source absorbed by the crop, per scenario the average net photosynthesis per MJ absorbed or emitted PAR light, referred to as light use efficiency (LUE) in its most usual unit, is calculated. Analysis of variance and *t*-test for differences were carried out in GenStat, 16th Edition.

## RESULTS

The tomato leaves reflected and transmitted 8 and 3% of PAR light, respectively. The greenhouse floor reflected on average ca. 40% of PAR light and the white plastic 75%. For the relatively small area of stem surface we assumed similar optics as for leaves.

**FIGURE 2:**
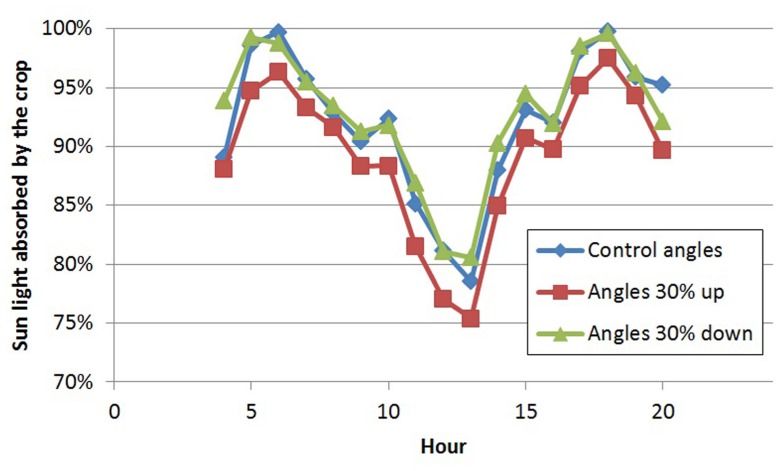
**Diurnal pattern of light absorption (% of incoming radiation) by the crop on day 180 for three scenarios of leaf angles**.

With the 3D structure of the measured crop, results of the scenarios on leaf angles showed an effect of maximally 3% relative to the control for most situations (**Table 1**). In the control situation, the rachis of mature leaves has an almost horizontal position (see indicated angle in **Figure 1C**), and the attached leaflets hung down by another 30–50°. Modeling a larger angle of the rachis relative to the main stem (scenario 1.1a) made the leaves point a little upward, which was detrimental for light interception (**Table 1**; **Figure 2**). Leaves that pointed more downward (scenario 1.1.c) did not increase light absorption of sun light compared to default, horizontal leaves. Averaged over the day, this scenario, however, showed absorption to decrease 2% around noon and increase 2% in morning and afternoon relative to default. For sun light the leaf angle changes, similar to light absorption, only decreased LUE for steeper leaves (scenario 1.1.a). The day average of LUE for absorbed light was relatively low as compared to other scenarios, and despite 20% higher photosynthetic potential in summer, due to the higher light levels which at noon (at 1400 μmol PAR m^-^^2^ s^-^^1^) resulted in light saturation.

**Table 1:** Light absorption (% of input) and light use efficiency (LUE) per unit absorbed or emitted light per mentioned light source of the tomato crop for the different scenarios. Within each group (1.1, 1.2, 1.3, 2, or 3) letters behind a mean indicate a significant difference between scenarios (*p* < 0.05). DM, dry matter.

Scenario	Property	Value changed	Light absorption (% of input)	LUE (g DM MJ^-1^ absorbed PAR)	LUE (g DM MJ^-1^ emitted PAR)
				**Sunlight (30% diffuse)**^#^
1.1.a	Leaf	+30%	90.2 ± 0.06a	3.08 ± 0.06	2.77 ± 0.05a
1.1.b	angle	0	93.1 ± 0.06b	3.10 ± 0.06	2.86 ± 0.05b
1.1.c		-30%	93.7 ± 0.06b	3.07 ± 0.06	2.90 ± 0.05b
				**HPS lamps only**
1.2.a		+30%	85.8 ± 0.8a	4.22 ± 0.07	3.62 ± 0.05
1.2.b		0	87.3 ± 0.6b	4.21 ± 0.06	3.67 ± 0.06
1.2.c		-30%	88.0 ± 0.3b	4.15 ± 0.06	3.65 ± 0.05
				**LED inter-lighting only**
1.3.a		+30%	93.7 ± 0.5	4.08 ± 0.06a	3.82 ± 0.06a
1.3.b		0	94.1 ± 0.5	4.09 ± 0.06a	3.84 ± 0.05a
1.3.c		-30%	93.5 ± 0.5	3.56 ± 0.05b	3.33 ± 0.05b
				**LED inter-lighting only**
2.a	Leaf	Toward path	96.0 ± 0.4a	3.11 ± 0.11a	2.98 ± 0.09a
2.b	direction	Parallel to slab	94.1 ± 0.3b	3.64 ± 0.05b	3.43 ± 0.04b
				**LED inter-lighting only**
3.a	LED light direction	+30^°^	92.0 ± 0.8a	3.98 ± 0.13a	3.66 ± 0.12a
3.b		+20^°^	95.5 ± 0.2b	4.14 ± 0.14a	3.95 ± 0.13b
3.c		+10^°^	94.5 ± 0.5ab	3.97 ± 0.06ab	3.75 ± 0.06ab
3.d		0^°^	94.1 ± 0.5ab	4.09 ± 0.07ab	3.84 ± 0.05ab
3.e		-10^°^	86.8 ± 0.7c	3.38 ± 0.06b	2.93 ± 0.05c
3.f		-20^°^	81.3 ± 1.0d	3.13 ± 0.06c	2.54 ± 0.04d
3.g		-30^°^	78.8 ± 1.3e	2.48 ± 0.05d	1.95 ± 0.04e

*^#^For sunlight scenarios a measured summer crop structure was used instead of the default winter structure*

For winter the measured crop structure had similar leaf angles, yet smaller LAI (2.7 vs. 3.4), longer internodes (0.11 vs. 0.08 m on average) and a 20% lower maximum photosynthesis relative to summer, and was used for HPS and LED scenarios. For HPS lighting, raising the leaves from -30 to +30% from default, decreased light absorption but hardly affected LUE (**Table 1**). The latter means that between these leaf angle scenarios the light-absorbing leaves all have a similar leaf age and thus similar photosynthetic potential. On the contrary, for the LED scenarios with horizontal and steeper leaves (scenarios 1.3.a,b) the increased absorption did not increase but lighting more upper leaves were illuminated as shown by increased LUE relative to down-hanging leaves (scenario 1.3.c).

Leaves oriented to the path (scenario 2.a) had only 2% more interception than leaves oriented toward the slab (**Table 1**), yet utilization per MJ emitted light was lower than leaves oriented parallel to the row and slab (scenario 2.b).

Directing LEDs more upward up to 20° (scenario 3.b) resulted in higher absorption of LED light by the crop and the highest LUE per MJ emitted light of all scenarios. In this scenario no increased loss of light to the sky was simulated since the LEDs were positioned rather low in the crop. Pointing the light more downward did dramatically decrease light interception (only 78% of input at scenario 3.g) and led to less photosynthesis due to the lower performance of the lower leaves (**Table 1**).

**FIGURE 3:**
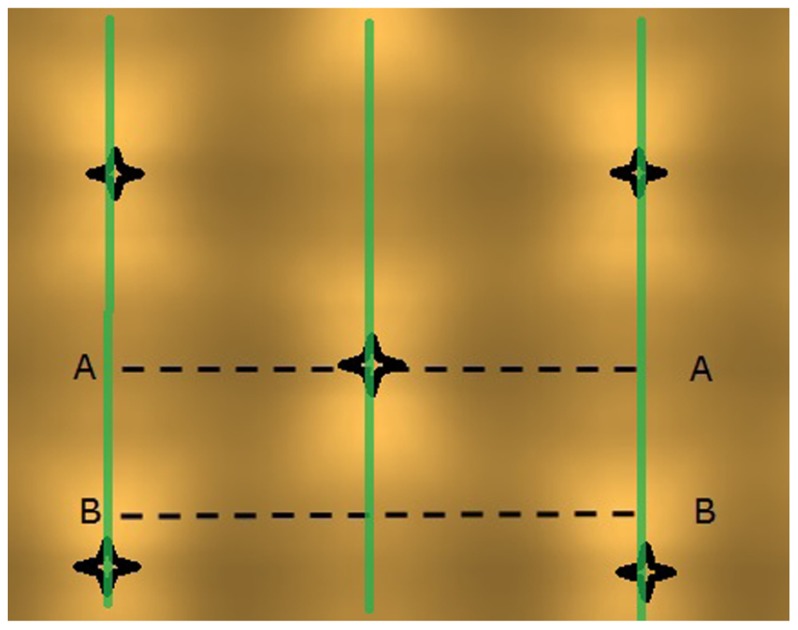
**Projected light pattern of HPS lamps without plants, on a horizontal plane at 2.5 m distance from the lamps, with sensor trajectories (A – A and B – B) indicated by dashed lines, lamp positions are shown by star-like symbols, and the double plant rows by green lines**.

**FIGURE 4:**
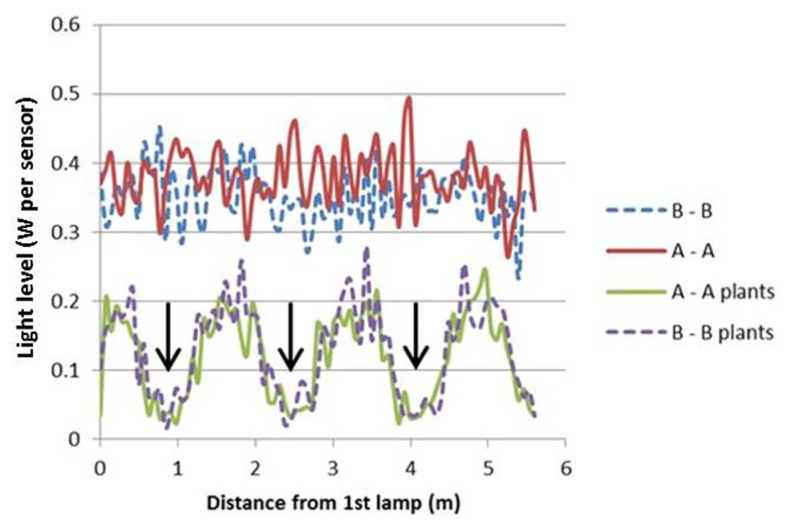
**Computed light intensity (W per sensor with 6 cm radius) at the trajectories of **Figure 3**, with and without light intercepting plants (plant positions indicated by arrows)**. For this simulation the HPS lamps had an arbitrary light output of 1000 W m^-^^2^.

The light model computed considerable horizontal differences in light intensity within the crop. For HPS lamps without plants, the visual rendering seemed to indicate large intensity differences in the area between lamps as shown by illumination of a horizontal plane (**Figure 3**), yet the sensed light level showed only modest differences (**Figure 4**). When plants are introduced in the model, the sensed light level between positions in and outside the plant row were large (**Figure 4**). Also for LEDs, after light penetration through the plant, hardly any light remained to illuminate the neighboring row (data not shown), suggesting each double plant row should contain a LED module for homogeneous illumination.

## DISCUSSION

A 3D model of light including leaf photosynthesis has seldom been used to predict effect of artificial light on LUE of the crop (Delepoulle et al., 2008; de Visser and Buck-Sorlin, 2011). The present, unique model indicates that HPS lamps result in a higher photosynthesis per unit absorbed light than LEDs. This is caused by the higher LUE of upper leaves, catching more light for HPS than for LEDs. Yet, per emitted MJ, LED lighting is more efficient than HPS lighting due to a higher fraction of absorbed light. Apart from HPS lamps illuminating the upper leaves with high photosynthetic capacity, an additional advantage of HPS relative to LED is that they combine PAR and heat radiation, thus supporting both assimilate supply by photosynthesis and temperature driven organ development. Despite the efficiency of LED light interception, their PAR emission should be accompanied by a certain level of heating, reducing their energy-saving advantages. Only a 3D model incorporating energy balance calculations can fully estimate the energy efficiency of a LED strategy.

Tilting the leaves upward decreased light interception of the downward light from sun and HPS lamps, but not for LEDs. Very probably leaves have less surface exposed to the sun, alike more erect leaves in arid, sunny climates that prevent excess light heating the leaves. The decreased light interception did not affect LUE because still the same leaves were illuminated, at similar photosynthetic potential. In contrast, light interception was not affected for LEDs when leaf angles were changed. Yet, when down by -30%, the lowest leaves were illuminated more which resulted in a lower computed LUE due to lower photosynthetic capacity of these leaves. The winter crop structure was different from summer structure in terms of internode length and leaf area, but not in terms of leaf angles. We expect that the longer internodes in winter do result in a wider spread of LED light.

The turning of leaves predominantly toward the path slightly increased LED light interception relative to default leaf positions, whereas leaves predominantly oriented parallel to the rows did not alter light interception relative to default. Leaves may orient toward LED light or turn away at too high light level (e.g., Trouwborst et al., 2011), thus impacting the interception of LED light.

For LEDs, the beaming angle had a strong effect on light absorption and LUE. The highest light interception was modeled at 20° up from horizontal position, and a significant decrease from 0° toward -30° angle due to loss to the ground. This effect may have been lower if the LEDs were positioned higher in the crop, showing the sensitivity of our results to the particular situation of LED placement and crop architecture. Placement of LEDs should be carefully planned, taking into consideration the given plant structure. A similar conclusion was drawn by Sarlikioti et al. (2011b) who observed strong effects of plant structure on light interception, in particular for internode lengths and leaf shape. The same authors found that leaves orientate themselves toward the path to intercept more light (Sarlikioti et al., 2011a), which agrees to our findings of a modest 2% light interception increase relative to orientation parallel to the plant row, as calculated for LED light.

Optimal lighting of leaves is driven by a combination of spatial emission pattern and crop characteristics such as structure and optical properties. We did not test the effect of the emitted light spectrum and its transformation (selective absorption, transmission, and reflection) as the light rays pass through the canopy. A recent extension of GroIMP, the GPUFlux light model (van Antwerpen et al., 2011), is a spectral Monte Carlo light tracer, which offers this possibility. The light tracer utilizes available computing resources through OpenCL. For each object, the model either computes a fully discretized absorption spectrum or several integrated weighted spectra, which are subsequently used in a photosynthesis model.

An important factor in optimization of light use is the positioning of light sources close to leaves with highest photosynthetic potential. It is standard practice that lamp power is accommodated such that the light level on nearby leaves is not at a saturating level but still in the linear trajectory of the light-response of photosynthesis. This is now also accommodated for LED modules when using interlighting, resulting in an average supplement of about 60 μmol PAR m^-^^2^ s^-^^1^ greenhouse. Whether this light amount will not lead to deleterious, high light levels on nearby leaves could be verified using the present model, ultimately leading to an advice to, e.g., use lower intensities and save energy. Yet, leaves can adapt their photosynthetic properties in response to their history of perceived local light, resulting, e.g., in sun-adapted leaves with high amounts of Rubisco and a high photosynthetic capacity. In a high-wired and dense crop, leaves normally decrease their photosynthetic capacity as well as their compensation point following adaptation to decreasing levels of perceived light lower in the canopy (Trouwborst et al., 2011). However, prolonged high light levels on aging cucumber leaves halted the decrease of photosynthetic capacity (Hovi-Pekkanen and Tahvonen, 2008). This is also aimed at with LED interlighting, yet this has to be confirmed experimentally. Maintenance of photosynthetic capacity would be another advantage of interlighting, adding to the lower light losses as shown in this study.

The computer graphical representation of tomato leaves that we used in our model was a combination of textured cylinders (petioles and rachies) and flat boxes (leaflets). This was clearly a simplification as tomato leaflets are in reality variously convexly or concavely curved surfaces: such curved surfaces will increase diffuse reflection and probably lead to better LUE in a dense canopy. Such an effect can at present not be considered by our model, but the representation of leaves as complexly curved surfaces is technically possible (Gerhard Buck-Sorlin and Michael Henke, unpublished work).

In conclusion, based on the present simulation study we would be able to give the following, tentative, recommendations to improve the efficacy of assimilation light in the greenhouse: LEDs should be preferred over HPS as the light interception efficiency is bigger; the crop’s LUE for HPS is higher than for LED due to lighting a higher fraction of leaves with higher photosynthetic capacity; light interception of LED interlighting is increased if LEDs are sufficiently high above the greenhouse floor and pointing slightly upward, thereby avoiding loss of light to the ground.

## Conflict of Interest Statement

The authors declare that the research was conducted in the absence of any commercial or financial relationships that could be construed as a potential conflict of interest.

## References

[B1] Buck-Sorlin G. H. Hemmerling R. Vos J. de Visser P. H. B. (2009). “Modelling of spatial light distribution in the greenhouse: description of the model,” in *Proceedings of the Third International Symposium on Plant Growth Modeling, Simulation, Visualization and Applications* Beijing 10.1109/PMA.2009.45

[B2] Buck-Sorlin G. H. Visser P. H. B. de Henke M. Sarlikioti V. Heijden G. W. A. M. van der Marcelis L. F. M. (2011). Towards a functional–structural plant model of cut-rose: simulation of light environment, light absorption, photosynthesis and interference with the plant structure. *Ann. Bot.* 108 1121–1134 10.1093/aob/mcr190 21856634 PMC3189845

[B3] Chelle M. Andrieu B. (1998). The nested radiosity model for the distribution of light within plant canopies. *Ecol. Model.* 111 75–91 10.1016/S0304-3800(98)00100-8

[B4] Cieslak M. Lemieux C. Hanan J. Prusinkiewicz P. (2008). Quasi-Monte Carlo simulation of the light environment of plants. *Funct. Plant Biol.* 35 837–849 10.1071/FP08082 32688836

[B5] Delepoulle S. Renaud C. Chelle M. (2008). “Improving light position in a growth chamber through the use of a genetic algorithm,” in *Artificial Intelligence Techniques for Computer Graphics, Studies in Computational Intelligence* Vol. 159/2008 (Berlin: Springer) 67–82

[B6] de Visser P. H. B. Buck-Sorlin G. H. (2011). *Modelling Spatial Light Distribution in Crops (in Dutch)*. GTB report 1104. Wageningen, UR Greenhouse Horticulture Netherlands

[B7] de Visser P. H. B. Buck-Sorlin G. H van der Heijden G. W. A. M. Marcelis L. F. M. (2012). A 3D model of illumination, light distribution and crop photosynthesis to simulate lighting strategies in greenhouses. *Acta Hortic.* 956 195–200

[B8] de Visser P. H. B. Heijden G. W. A. M. van der Heuvelink E. Carvalho S. M. P. (2007). “Functional-structural modelling of chrysanthemum,” in *Functional-Structural Plant Modelling in Crop Production* eds Vos J. Marcelis L. F. M. de Visser P. H. B. Struik P. C. Evers J. B. (Dordrecht: Springer) 199–208 10.1007/1-4020-6034-3_17

[B9] Hovi-Pekkanen T. Tahvonen R. (2008). Effects of interlighting on yield and external fruit quality in year-round cultivated cucumber. *Sci. Hortic.* 761 183–191

[B10] Goudriaan J van Laar H. H. (1994). *Modelling Potential Crop Growth Processes.* Dordrecht: Kluwer Academic Publishers. 10.1007/978-94-011-0750-1

[B11] Kahlen K. Stützel H. (2011). Modelling photo-modulated internode elongation in growing glasshouse cucumber canopies. *New Phytol.* 190 697–708 10.1111/j.1469-8137.2010.03617.x 21251000

[B12] Kahlen K. Wiechers D. Stützel H. (2008). Modelling leaf phototropism in a cucumber canopy. *Funct. Plant Biol.* 35 876–884 10.1071/FP08034 32688839

[B13] Kim S-H. Lieth J. H. (2003). A coupled model of photosynthesis, stomatal conductance and transpiration for a rose leaf (*Rosa hybrida* L.). *Ann. Bot.* 91 771–781 10.1093/aob/mcg080 12730065 PMC4242386

[B14] Kniemeyer O. (2008). *Design and Implementation of a Graph Grammar Based Language for Functional-Structural Plant Modelling*. Doctoral dissertation, Fakultät für Mathematik, Naturwissenschaften und Informatik, Brandenburg University of Technology Cottbus

[B15] Sarlikioti V. Visser P. H. B de Marcelis L. F. M. (2011a). Exploring the spatial distribution of light absorption and photosynthesis of canopies by means of a functional-structural plant model. *Ann. Bot.* 107 875–883 10.1093/aob/mcr006 21355008 PMC3077986

[B16] Sarlikioti V. Visser P. H. B. de Buck-Sorlin G. H Marcelis L. F. M. (2011b). How plant architecture affects light absorption and photosynthesis in tomato: towards an ideotype for plant architecture using a functional-structural plant model. *Ann. Bot.* 108 1065–1073 10.1093/aob/mcr221 21865217 PMC3189847

[B17] Trouwborst G. Hogewoning S. W. Harbinson J Ieperen W. van (2011). The influence of light intensity and leaf age on the photosynthetic capacity of leaves within a tomato canopy. *J. Hortic. Sci. Biotechnol.* 86 403–407

[B18] Trouwborst G. Oosterkamp J. Hogewoning S. W. Harbinson J Ieperen W. van (2010). The responses of light interception, photosynthesis and fruit yield of cucumber to LED-lighting within the canopy. *Physiol. Plant.* 138 289–300 10.1111/j.1399-3054.2009.01333.x 20051030

[B19] van Antwerpen D. van der Heijden G. W. A. M. Marcelis L. F. M. de Visser P. B. Buck-Sorlin G. H. Jansen E. (2011). “High performance spectral light transport model for agricultural applications,” in *Proceedings of the ACM SIGGRAPH/EUROGRAPHICS Conference on High Performance Graphics 2011* eds Dachsbacher C. Mark W. Pantaleoni J. (Vancouver: Eurographics Association)

[B20] Veach E. (1997). *Robust Monte Carlo Methods for Light Transport Simulation*. Ph.D. Dissertation, Stanford University Standford Available at:

[B21] Vos J. Evers J. B. Buck-Sorlin G. H. Andrieu B. Chelle M de Visser P. H. B. (2010). Functional–structural plant modelling: a new versatile tool in crop science. *J. Exp. Bot.* 61 2101–2115 10.1093/jxb/erp345 19995824

[B22] Wiechers D. Kahlen K. Stützel H. (2011). Dry matter partitioning models for the simulation of individual fruit growth in greenhouse cucumber canopies. *Ann. Bot*. 108 1075–1084 10.1093/aob/mcr150 21715366 PMC3189842

